# Letter to editor: comment on the study “Català I, Roldán H, Fernández-Carballal C, Domínguez-Alonso C, Álvarez-Galovich L, Godino Ó. A new hernia blocking system prevents lumbar disc herniation recurrence and disc degeneration: 2-year results of a multicentric clinical investigation. **Brain Spine**. 2025;6:105898. https://doi.org/10.1016/j.bas.2025.105898

**DOI:** 10.1016/j.bas.2026.106001

**Published:** 2026-03-03

**Authors:** Sourabh Zambre, Varidh Katiyar, Alok Umredkar

**Affiliations:** Department of Neurosurgery, All India Institute of Medical Sciences, Nagpur, India

Letter to the Editor,

We read the article “A new hernia blocking system prevents lumbar disc herniation recurrence and disc degeneration: 2-year results of a multicentric clinical investigation” by Ignasi Catala et al., with great intrigue (Catala' I et al., 2025). Lumbar disc prolapse extracts a heavy toll on the patient and healthcare apparatus, with a huge economic burden for treatment and loss of wages. It affects the entire family and society at large. Recurrent disc prolapse is a well-known risk of disc prolapse surgery, with reported rates ranging from 3 to 25 %, which increases steadily with longer follow-up. We appreciate the authors for evaluating a new device that addresses this issue by mechanically maintaining the nucleus pulposus in its physiological position.

The authors have acknowledged that the primary limitation of the study is the lack of a direct control group. To address this, they have compared their participants with a control group from Thome et al., with similar participant characteristics (Thome C et al., 2018). In this RCT by Thome et al., the authors compared a similar annular closure device, Barricaid with controls. In our opinion, the authors should have discussed the safety and efficacy of their implant with respect to the intervention arm (Barricaid) of Thome et al., in addition to the control group. It would have been more appropriate for the authors to have used a historical control cohort from the same institute to minimise biases related to patient, institute and surgeon-related factors. Additionally, despite stating the use of external control in the methodology, a formal statistical test to compare the outcomes with the control group has not been attempted. However, in the discussion and conclusion section, the authors have stated their findings in terms of ‘reduction of rLDH with the use of their device’, which might seem inappropriate without a statistical comparison with a control group.

It is also interesting to note that even when the Barricaid system was found to be effective in the RCT by Thome et al., and in a meta-analysis by Miller et al. (including Thome et al. and three smaller controlled studies), it has not gained widespread acceptance among spine surgeons worldwide (Miller LE et al., 2020). The most significant deterrent in a low- and middle-income country could be its cost. Also, surgeons might be biased against placing an additional foreign body inside the surgical field, anticipating device-related complications. Firstly, the mesh used may act as a source of infection or bio membrane formation, which may be resistant to systemic antibiotic coverage. Secondly, it may increase scarring and fibrosis, which in turn may cause stretching or compression of the traversing root. This side effect can be anticipated based on the mechanism of action of meshes used in abdominal hernia repairs, where fibrosis is the intended effect. In the event of inadvertent dural puncture during surgery, this scarring effect may extend to involve the cauda equina, resulting in additional clinical deficit. Thirdly, if the anchor loosens, there can be migration of the device, causing cauda equina syndrome. Additionally, it is also unclear how the device would perform in high-risk cases like patients with high BMI, immunocompromised status, osteoporosis, axial spondyloarthritis, spondylolisthesis, lumbar canal stenosis and traumatic aetiology.

These are some of the concerns that may deter most surgeons from accepting such devices in routine clinical practice. This may gradually change as more literature is available regarding the safety of such devices. Practice trends change based on social proofing in the geographical and economic context.

Future research could be envisaged comparing the different available devices and suturing methods for mechanical buttressing of the open annulus defect (Ying Y et al., 2023). Although it's not a new concept, the author's efforts to evaluate a new device, increasing the choices available to both the patients and surgeons, may help in bringing down the cost of such implants in the future.

## Declaration of competing interest

The authors declare that they have no known competing financial interests or personal relationships that could have appeared to influence the work reported in this paper.
